# Cross-Border Movement of Highly Drug-Resistant *Mycobacterium tuberculosis* from Papua New Guinea to Australia through Torres Strait Protected Zone, 2010–2015

**DOI:** 10.3201/eid2503.181003

**Published:** 2019-03

**Authors:** Arnold Bainomugisa, Sushil Pandey, Ellen Donnan, Graham Simpson, J’Belle Foster, Evelyn Lavu, Stenard Hiasihri, Emma S. McBryde, Rendi Moke, Steven Vincent, Vitali Sintchenko, Ben J. Marais, Lachlan J.M. Coin, Christopher Coulter

**Affiliations:** Pathology Queensland, Brisbane, Queensland, Australia (A. Bainomugisa, S. Pandey, C. Coulter);; The University of Queensland, Brisbane (A. Bainomugisa, L.J.M. Coin);; Queensland Department of Health, Brisbane (E. Donnan, C. Coulter);; Cairns Tuberculosis Unit, Cairns, Queensland, Australia (G. Simpson, S. Vincent);; James Cook University, Townsville, Queensland, Australia (J. Foster, E.S. McBryde);; Central Public Health Laboratory, Port Moresby, Papua New Guinea (E. Lavu);; Western Province Health Office, Daru, Papua New Guinea (S. Hiasihri);; Port Moresby General Hospital, Port Moresby (R. Moke);; Westmead Hospital, Sydney, New South Wales, Australia (V. Sintchenko);; The University of Sydney, Sydney (V. Sintchenko, B.J. Marais)

**Keywords:** whole-genome sequencing, tuberculosis, MDR TB, Torres Strait, Papua New Guinea, Australia, tuberculosis and other mycobacteria, mycobacteria, bacteria, *Mycobacterium tuberculosis*, antimicrobial resistance, drug resistance, cross-border movement, transmission, Beijing sublineage 2.2.1.1, isoniazid, ethionamide, TB

## Abstract

In this retrospective study, we used whole-genome sequencing (WGS) to delineate transmission dynamics, characterize drug-resistance markers, and identify risk factors of transmission among Papua New Guinea residents of the Torres Strait Protected Zone (TSPZ) who had tuberculosis diagnoses during 2010–2015. Of 117 isolates collected, we could acquire WGS data for 100; 79 were Beijing sublineage 2.2.1.1, which was associated with active transmission (odds ratio 6.190, 95% CI 2.221–18.077). Strains were distributed widely throughout the TSPZ. Clustering occurred more often within than between villages (p = 0.0013). Including 4 multidrug-resistant tuberculosis isolates from Australia citizens epidemiologically linked to the TSPZ into the transmission network analysis revealed 2 probable cross-border transmission events. All multidrug-resistant isolates (33/104) belonged to Beijing sublineage 2.2.1.1 and had high-level isoniazid and ethionamide co-resistance; 2 isolates were extensively drug resistant. Including WGS in regional surveillance could improve tuberculosis transmission tracking and control strategies within the TSPZ.

Tuberculosis (TB) is the leading infectious cause of death globally (*1*). To reduce the burden of TB, many countries committed to achieving a 90% reduction in TB incidence by 2035 as part of the End TB Strategy (*2*). Australia has already achieved preelimination targets (<10 cases/1 million population) in the nonindigenous, Australia-born population (*3*). However, achieving TB elimination in Australia remains a daunting challenge, given high population mobility, increased importation of TB cases from high-incidence settings, and cross-border spread from neighboring countries, such as Papua New Guinea (*4*). In 2013, ≈90% of the TB cases reported in Australia were in persons born overseas (*3*). The state of Queensland has one of the lowest TB notification rates in Australia (4.0 cases/100,000 population) (*5*); the Western Province of neighboring country Papua New Guinea has a significantly higher incidence, estimated at 2,901 cases/100,000 population at the provincial capital, Daru (*6*,*7*).

Daru General Hospital (Port Moresby, Papua New Guinea) is the main health center that offers TB health services to residents of Western Province, including those in the surrounding areas, such as the Torres Strait Protected Zone (TSPZ). The TSPZ is an area where free bidirectional cross-border movement (without passports or visas) is permitted for purposes of traditional customs and economic activities (Figure 1); the zone was created with the signing of the 1978 Torres Strait Treaty by Papua New Guinea and Australia. This area contains a number of Papua New Guinea villages and 14 Australia island communities (estimated population 1,526) that are part of Queensland (*9*). Cross-border movement of populations within the TSPZ provides a potential route of entry of *Mycobacterium tuberculosis* into northern Queensland and its spread elsewhere in Australia.

**Figure 1:**
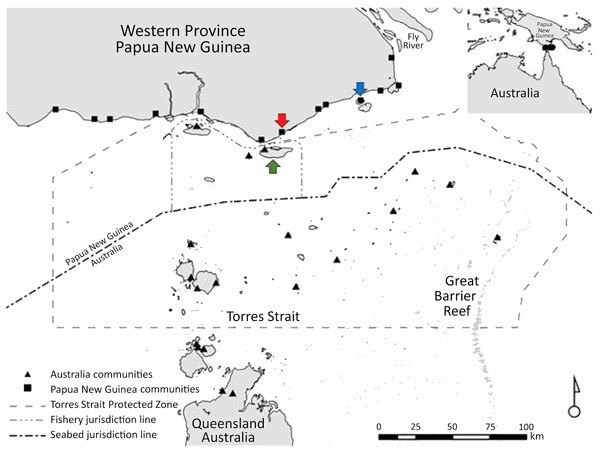
Communities within and boundaries of Torres Strait Protected Zone. Green arrow indicates Saibai Island, Australia; red arrow Mabaduan village, Papua New Guinea; and blue arrow Daru Island, Western Province Provincial Capital, Papua New Guinea. Inset depicts location of Torres Strait Protected Zone between Australia and Papua New Guinea. Operational details on how the 1978 Torres Strait Treaty functions can be accessed online (https://dfat.gov.au/geo/torres-strait/Documents/torres-strait-guidelines.pdf). Map obtained with permission from Butler JRA, Tawake A, Skewes T, McGrath V. Integrating traditional ecological knowledge and fisheries management in the Torres Strait, Australia: the catalytic role of turtles and dugong as cultural keystone species. Ecology and Society. 2012;17:34 (*8*).

In 2012, an Australia resident of the TSPZ was reported to have pulmonary TB resistant to streptomycin only. After initial improvement with standard therapy, the patient deteriorated clinically. Rifampin resistance was detected by Xpert MTB/RIF assay (Cepheid, http://www.cepheid.com), and multidrug-resistant (MDR) TB was confirmed after positive culture and drug susceptibility testing. The mycobacterial interspersed repetitive unit 24 (MIRU-24) profile of the new isolate differed from the initial isolate and was characteristic of TB strains in Western Province, indicating a possible reinfection with an MDR TB isolate (*10*). This incident was the first notified case of MDR TB in a citizen of Australia in the Torres Strait Islands. During outbreak epidemiologic investigations, 3 additional MDR TB diagnoses were made in citizens of Australia who were current or previous residents of the TSPZ. These 4 cases were epidemiologically linked through a network of close contacts. At the time of diagnosis, 2 of the patients were living in different Queensland cities distant from the TSPZ.

We sought to use whole-genome sequencing (WGS) to determine the genomic relationship between these isolates and the MDR TB isolates from Papua New Guinea as proof of principle for transmission of MDR TB through the TSPZ. The discriminatory power of WGS enables the delineation of TB transmission with a higher resolution than conventional genotyping (*11*,*12*). The aim of this study was to use WGS and epidemiologic data to determine strain diversity within the TSPZ, characterize geno-resistance markers, and identify potential risk factors for transmission.

## Methods

### Strain Selection

All patients receiving TB diagnoses in Queensland (including Papua New Guinea citizens receiving TB diagnoses in Queensland clinics in the TSPZ) must be reported to the Queensland Department of Health in Brisbane. In this study, we included isolates from Papua New Guinea citizens residing in the TSPZ who received TB diagnoses in the TSPZ during January 1, 2010–December 31, 2015, which we refer to as cross-border TB isolates. We extracted demographic and clinical data from the Queensland Notifiable Conditions System on April 6, 2016. We included 1 patient given a TB diagnosis previously in Papua New Guinea who subsequently sought treatment in the TSPZ for extensively drug-resistant (XDR) TB; we also included the 4 previously mentioned MDR TB cases in residents of Australia because these infections were linked to the TSPZ. During the study period, a small number of drug-susceptible TB cases (n = 14) were reported among Australia citizens residing in the TSPZ, but we did not include these cases in this study.

### Drug Susceptibility Testing and Genotyping

We performed *M. tuberculosis* culture, species identification, genotyping, and drug susceptibility testing at Queensland Mycobacterium Reference Laboratory (Pathology Queensland, Brisbane). MIRU-24 or MIRU-15 results obtained as previously described (*13*) were available for most isolates. We determined phenotypic susceptibility to first- and second-line drugs as previously described (*7*). On a case-by-case basis, we used SENSITITRE Microbroth Dilution Method (Trek Diagnostic Systems, https://www.thermofisher.com) to resolve differences between resistance mutations detected and phenotypic drug susceptibility test results.

### WGS Analysis

We retrieved *M. tuberculosis* isolates from −80°C storage, cultured them on Lowenstein–Jensen medium, and extracted isolate DNA using an organic enzymatic method (*7*). We performed WGS at the Australia Genome Research Facility (Brisbane) using Illumina HiSeq 2000 (https://www.illumina.com) for paired-end reads, checked the quality of reads using FastQC version 0.11.2 (http://www.bioinformatics.babraham.ac.uk/projects/fastqc), and trimmed using trimmomatic version 0.27 (*14*). We mapped reads to reference genome *M. tuberculosis* H37Rv (GenBank accession no. NC_000962.3) using BWA-MEM (https://arxiv.org/abs/1303.3997) and used GATK UnifiedGenotyper (*15*) to call single-nucleotide polymorphisms (SNPs) and small insertion/deletions (indels). We selected the SNPs and small indels with >10 times read depth, 80% allele frequency, and >10-bp difference between neighboring SNPs and indels. Using SnpEff version 4.1 (*16*), we annotated high-quality SNPs and indels. We included for analysis mutations in all known genes (including regulatory genes) that conferred resistance to TB drugs (Appendix Table 1) and excluded mutations in repetitive regions, such as the *PE* and *PPE* gene family regions. We submitted raw reads in the form of FASTQ files to the Sequence Read Archive (project file no. PRJNA401368). Scripts of the raw sequence data are available (https://github.com/arnoldbaino/Daru_scripts).

### Phylogenetics and Bayesian Coalescent Analysis

We used data sets from 2 previous independent WGS studies, PRJEB7281 and PRJEB2358 (*17*), as TB global representatives in phylogenetic analysis. Using concatenated SNP alignment, we constructed a maximum-likelihood phylogenetic tree with RAxML version 7.4.2 (*18*). We used a general time-reversible model, with rate heterogeneity accommodated by using discrete rate categories (i.e., GTRCAT algorithm), with 1,000 bootstraps and visualized using FigTree version 1.4.2 (http://tree.bio.ed.ac.uk/software/figtree). We performed molecular dating of the Beijing lineage isolates using BEAST version 1.8.2 (*19*), as previously described (*7*).

### Transmission Assessment

Using ape library in R statistical package (http://cran.r-project.org), we calculated pairwise SNP differences (excluding SNPs in known drug-resistance genes) between isolates. We assessed SNP differences by lineage and compared these differences between different localities to set a threshold as a measure of putative transmission. We constructed genomic clusters using a median-joining network (Network version 5, http://www.fluxus-engineering.com/sharenet.htm). We structured links within genomic clusters assuming the existence of linear transmission among isolates from the same locality and putative transmission among isolates from different localities (if the SNP difference was within the limit of the threshold).

### Statistical Analysis and Ethics

We compared patient characteristics using Fisher exact test. We performed univariate and multivariable logistic regression analyses to evaluate associations between patient characteristics and genomic clusters as outcome variables to infer transmission. We used R statistical package for all statistical analyses. This study was approved by the institutional review board of the University of Queensland (Brisbane, Queensland, Australia; approval no. HREC2015000572) and, as required by the Public Health Act of 2005, by Queensland Health (approval no. RD006697).

## Results

**Figure 2:**
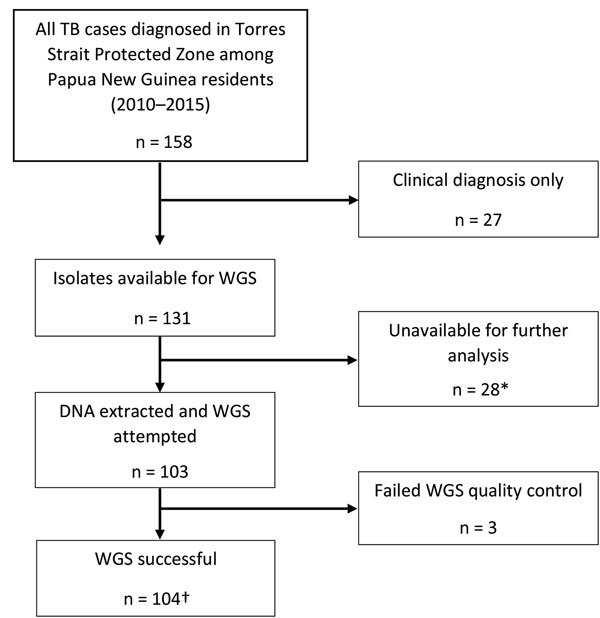
Flow diagram of included *Mycobacterium tuberculosis* isolates from Papua New Guinea citizens residing in Torres Strait Protected Zone, 2010–2015. *Isolates unable to grow or were contaminated. †Included were 4 additional isolates among Queensland residents that were a part of an epidemiologic cluster linked to the Torres Strait Protected Zone. TB, tuberculosis; WGS, whole-genome sequencing.

In total, 134 Papua New Guinea citizens were reported to have TB in the TSPZ during the study period (Figure 2), and 117 (87.3%) had culture-confirmed disease. We acquired and successfully sequenced 100 (85.5%) of 117 isolates from these citizens plus 4 isolates from the Australia citizens who resided or previously resided in the TSPZ. Isolates were sequenced with a mean coverage depth of 74 times (range 42–110 times) and mean coverage breadth of 98.5% (range 96.3%–99.8%). MIRU profiles were available for 98 of 104 patients (Appendix Table 2). Sequence comparisons revealed a median SNP difference of 31 (interquartile range [IQR] 24–38) for isolates with the same MIRU profile and 1,101 (IQR 1,066–1,119) for isolates with different MIRU profiles (Appendix Figure 1). SNP differences between all possible pairs within the data set were bimodal; 2 large peaks represent 2 different lineages. The possibility of cross-contamination among isolates with no SNP differences was negligible because specimens were processed and their DNA sequenced on different days. Of the 104 isolates sequenced, phylogenetic analysis revealed that 83, including the 4 MDR TB isolates from Australia citizens, were part of the modern Beijing sublineage 2.2.1.1 and 21 the Euro-American lineage, which consisted of 6 different sublineages (Appendix Table 3, Figure 2). Although the isolates were widely distributed throughout the TSPZ, 50% originated from a single Papua New Guinea village, Mabadauan (Appendix Figure 3).

Evolutionary analysis of the Beijing sublineage 2.2.1.1 isolates revealed that 59% (49/83) were related to clades previously identified in nearby Daru Island (Appendix Figure 4) (*7*) and 41% were a part of unique clades. The isolates from the Australia residents were clade C, which we inferred to have emerged in the 1980s.

**Figure 3:**
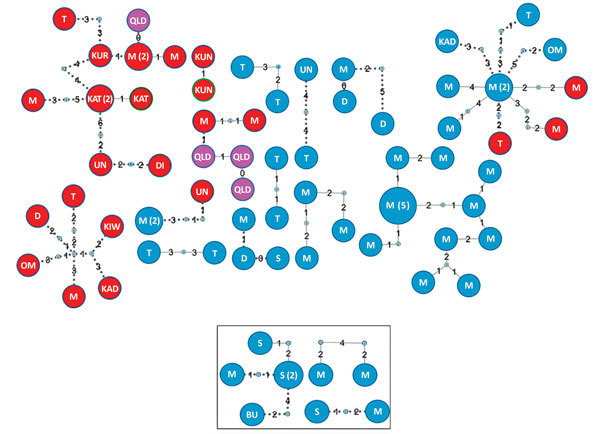
Genomic clusters of highly drug-resistant tuberculosis cases among Papua New Guinea and Australia citizens residing or previously residing in the Torres Strait Protected Zone, 2010–2015, by lineage. The top shows the Beijing lineage, and the box at bottom shows the Euro-American lineage. Each circle represents >1 isolate. Larger circles represent >2 isolates with identical sequences; the number of specimens with identical sequences are indicated in parentheses. Details indicated with each circle are locality (initials; QLD residents shaded in pink), drug susceptibility (blue shading with blue outline), and drug resistance (multidrug resistance is red shading with blue outline, extreme drug resistance is red shading with green outline). Solid lines illustrate plausible transmission links among isolates from the same locality, and broken lines represent plausible transmission links among strains from different localities. Small nodes between lines represent unidentified ancestral isolates. Number on lines represents single-nucleotide polymorphism differences between isolates. BU, Buji; D, Daru; DI, Dimiri; KAD, Kadawa; KAT, Katatai; KIW, Kiwia; KUN, Kunini; KUR, Kurunti; M, Mabadauan; OM, Old Mawata; QLD, Queensland; S, Sigabadaru; T, Ture Ture; UN, unknown.

To determine links between isolates from the same and different localities, especially isolates from villages with high numbers of isolates from different lineages, we deduced a threshold of 8 SNPs to distinguish transmission links (Appendix Figure 5). We found 17 genomic clusters (14 Beijing lineage, 3 Euro-American lineage) constituting 74 isolates (65 Beijing lineage, 9 Euro-American lineage) among PNG citizens (Figure 3). In total, 89% (34/38) of isolates from Mabadauan (identified as Beijing lineage) and 100% (4/4) of isolates from Sigabadaru (characterized as Euro-American lineage) clustered. More cases (36/41) formed genomic clusters in 2011 than in any other year, indicative of enhanced transmission leading up to this time point (Appendix Figure 6). The number of isolates from the same locality that formed genomic clusters (41 Beijing, 5 Euro-American) was significantly higher than the number that formed among isolates from different localities (24 Beijing, 4 Euro-American; p = 0.0013). The median SNP difference was 2 (IQR 1–3) among genomic clusters from the same locality and 4 (IQR 2–6) among genomic clusters from different localities. Of the 4 Australia cases, 3 formed 1 MDR TB cross-border genomic cluster having 1 SNP difference and a 15-month difference in sample collection dates. The fourth isolate was linked to another MDR TB cross-border cluster with no SNP differences and a 5-month difference in sample collection dates, suggesting 2 independent episodes of cross-border transmission into Australia citizens.

Most cross-border TB isolates in Papua New Guinea residents (74%, 74/100) were found in young persons (<35 years of age) (Table 1), and male and female sexes were equally represented. Only 1 (1.4%) of 74 patients tested had HIV co-infection. No major differences in characteristics were detectable between patients infected with the Beijing and Euro-American lineage, except for drug resistance, which was strongly associated with the Beijing lineage (p<0.001).

**Table 1:** Characteristics of Papua New Guinea residents infected with cross-border TB isolates, Torres Strait Protected Zone, 2010–2015, by lineage*

Characteristic	Total no. (%)	Lineage		p value†
Beijing	Euro-American
Age, y				0.739
<15	19 (19.0)	15	4	
15–24	31 (31.0)	26	5	
25–34	24 (24.0)	19	5	
35–49	18 (18.0)	13	5	
>50	8 (8.0)	6	2	
Year				0.324
2010	29 (29.0)	22	7	
2011	42 (42.0)	33	9	
2012	13 (13.0)	12	1	
2013	2 (2.0)	1	1	
2014	8 (8.0)	5	3	
2015	6 (6.0)	6	0	
Sex				0.876
F	45 (45.0)	35	10	
M	55 (55.0)	44	11	
Sputum smear				0.599
Negative	46 (46.0)	34	12	
Positive	54 (54.0)	45	9	
HIV status				0.234
Unknown	26 (26.0)	20	6	
Negative	73 (73.0)	59	14	
Positive	1 (1.0)	0	1	
Previous TB episode				0.653
No	87 (87.0)	68	19	
Yes	13 (13.0)	11	2	
Diagnostic delay, d				0.484
0–30	53 (53.0)	44	9	
31–60	18 (18.0)	14	4	
>61	29 (29.0)	21	8	
MDR or XDR				<0.001
No	69 (69.0)	48	21	
Yes	31 (31.0)	31	0	
Type of TB				0.741
Pulmonary	53 (53.0)	43	10	
Extrapulmonary	7 (7.0)	5	2	
Both	40 (40.0)	31	9	
Started TB treatment				0.472
No	22 (22.0)	15	7	
Yes	78 (78.0)	64	14	
TB treatment outcome				0.216
Transferred or lost to follow-up	66 (66.0)	52	14	
Cure or complete treatment	22 (22.0)	15	7	
Defaulted	3 (3.0)	3	0	
Died	9 (9.0)	9	0	

*MDR, multidrug resistant; TB, tuberculosis; XDR, extensively drug resistant. †p value calculated by using Fisher exact test.

Univariate analysis revealed that the Beijing lineage (odds ratio [OR] 6.190, 95% CI 2.221–18.077; p<0.0005) and resistance to first- and second-line TB drugs (OR 4.677, 95% CI 1.452–20.943; p = 0.019) were strongly associated with transmission (Table 2). In multivariate logistic analysis, we adjusted for these 2 characteristics, and this analysis showed the Beijing lineage was associated with transmission (adjusted OR 4.484, 95% CI 1.526–13.891). The HIV-infected patient was not part of a cluster.

**Table 2:** Characteristics associated with *Mycobacterium tuberculosis* isolate clustering among Papua New Guinea residents, Torres Strait Protected Zone, 2010–2015*

Characteristic	No. clustered/total no.	OR (95% CI)	p value	aOR (95% CI)†	p value
Beijing lineage	74/100				
No	9/21	Referent		Referent	
Yes	65/79	6.190 (2.221–18.077)	0.0005	4.484 (1.526–13.891)	0.007
Age, y					
<15	14/19	Referent		Referent	
15–24	25/31	1.488 (0.369–5.841)	0.565	1.298 (0.291–5.614)	0.725
25–34	19/24	1.357 (0.320–5.783)	0.673	1.242 (0.260–5.981)	0.782
35–49	12/18	0.714 (0.166–2.955)	0.641	0.722 (0.148–3.415)	0.679
>50	4/8	0.357 (0.059–2.016)	0.241	0.262 (0.035–1.785)	0.173
Sex					
F	34/45	Referent		Referent	
M	40/55	0.862 (0.343–2.118)	0.748	0.856 (0.311–2.284)	0.757
Sputum smear					
Negative	32/46	Referent		Referent	
Positive	42/54	1.531 (0.624–3.808)	0.352	1.147 (0.423–3.095)	0.784
Case type					
New case	63/87	Referent		Referent	
Recurrent	11/13	2.095 (0.513–14.183)	0.358	1.447 (0.270–11.282)	0.684
Diagnostic delay, d					
0–30	40/53	Referent		Referent	
31–59	13/18	0.845 (0.260–3.033)	0.784	1.042 (0.290–4.154)	0.95
>60	21/29	0.853 (0.308–2.455)	0.761	1.157 (0.380–3.743)	0.799
MDR or XDR					
No	46/69	Referent		Referent	
Yes	28/31	4.677 (1.452–20.943)	0.019	2.774 (0.779–13.123)	0.143
Treatment outcome					
Cure, treatment completed	13/22	Referent		Referent	
Transferred, lost to follow-up	50/66	2.163 (0.767–6.006)	0.138	1.794 (0.584–5.407)	0.298
Defaulted	2/3	NA	NA	NA	NA
Died	9/9	NA	NA	NA	NA

*aOR, adjusted OR; MDR, multidrug resistant; NA, not applicable (large value); OR, odds ratio; XDR, extensively drug resistant. †Adjusted for Beijing lineage and drug resistance (MDR or XDR).

Phenotypic drug susceptibility testing of all isolates showed that 53 (51.0%) were susceptible to all oral first-line drugs tested (Appendix Table 4); 1 showed streptomycin monoresistance. Drug-resistance patterns observed included 13.5% (14/104) with isoniazid and streptomycin co-resistance 31.7% (33/104) with MDR (1 pre-XDR with fluoroquinolone resistance), and 1.9% (2/104) with XDR. In 2010 and 2011, eleven TB cases were MDR or XDR (Appendix Figure 6). All MDR and XDR isolates, including the isolates from the 4 Australia citizens, belonged to clades C and D of Beijing sublineage 2.2.1.1.

**Figure 4:**
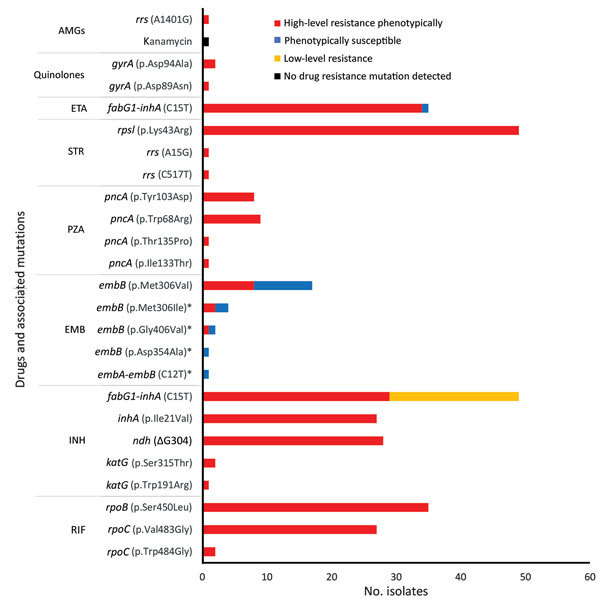
Correlation of phenotypic and genotypic drug resistance among modern Beijing isolates from Papua New Guinea and Australia citizens residing or previously residing in the Torres Strait Protected Zone, 2010–2015. No resistance mutation detected in 1 kanamycin-resistant strain, despite targeted sequencing of *rrs* and *eis* genes. *Pairs of co-occurring mutations observed within the same isolates were *embA*-*embB* (C12T) with *embB* (p.Asp354Ala) and *embB* (p.Gly406Val) with *embB* (p.Met306Ile). AMGs, aminoglycosides; EMB, ethambutol; ETA, ethionamide; INH, isoniazid; PZA, pyrazinamide; RIF, rifampin; STR, streptomycin.

Phenotypic and genotypic resistance profiles correlated, except for ethambutol (Figure 4). Of the 35 MDR and XDR isolates, which were also tested for second-line drug susceptibility, all carried the *fabG1*-*inhA* mutation (C15T), and 28 (80%) had high-level (0.4 µg/mL) isoniazid resistance. In total, 27 of these 28 had both *ndh* (ΔG304) and *inhA* (p.Ile21Val) mutations, and 1 had a *katG* (p.Trp191Arg) mutation. All the rifampin-resistant isolates had the same *rpoB* mutation (p.Ser450Leu), and 29 (82.9%) of 35 had compensatory mutations in the *rpoC* gene (27 p.Val483Gly and 2 p.Trp484Gly). Of note, nearly all isolates with compensatory *rpoC* mutations (28/29) and isolates with the *fabG1*-*inhA* mutation (48/49) were part of a genomic cluster. Of the 2 isolates with the p.Asp94Ala *gyrA* mutation conferring fluoroquinolone resistance, 1 was XDR; this isolate had pan–second-line injectable resistance associated with an *rrs* (A1401G) mutation. The second XDR isolate (from a patient previously treated in Papua New Guinea) had a p.Asp89Asn *gyrA* mutation and phenotypic kanamycin resistance without a detected mutation conferring aminoglycoside resistance. Mutations in genes potentially conferring resistance to newly available agents were not identified.

## Discussion

We characterized *M. tuberculosis* strain diversity, transmission dynamics, and drug-resistance profiles among cross-border isolates from TB patients residing in the TSPZ. This region is known for its sparse population, unique geography, and ethnic and social diversity, all of which can make disease surveillance difficult. The identification of drug-resistant isolates in every studied year and evidence of cross-border links underscore the risk for cross-border importation of drug-resistant TB with the potential to spread. Studies conducted in other parts of Australia suggest limited local TB transmission, despite a high proportion of imported TB cases (*20*,*21*), but those findings mainly reflected occurrences in urban areas. Residents of and visitors to the TSPZ might be vulnerable to community outbreaks; cultural and family relationships were associated with TB transmission in studies of community outbreaks within Australia among indigenous populations (*22*). We show that most transmission in the TSPZ occurred during local community outbreaks, as demonstrated by the high genomic clustering among patients from the same locality. With the economic growth anticipated in this region (*23*), interactions between Australia and Papua New Guinea populations could increase and affect disease notification trends.

The Beijing sublineage 2.2.1.1 dominated among isolates, and the Euro-American sublineage isolates were diverse. In a previous report, we attributed isolates from the same Beijing sublineage responsible for a large MDR TB outbreak on Daru Island, which is ≈50 km east of the outer islands of the TSPZ (*7*). The MDR TB outbreak on Daru Island was unprecedented in scale (*24*), but no published information described the extent of this outbreak beyond the shores of Daru Island.

In our study, most cases were identified to have come from Mabadauan, indicating that this setting could have a higher migratory rate than other TSPZ villages. The high number of drug-resistant TB cases from Mabadauan suggests this setting could be another hotspot for MDR TB transmission. Control of MDR TB transmission with effective clinical and public health–related interventions in this remote setting is urgently needed. Only 59% of Beijing lineage isolates from the TSPZ were part of clades previously identified on Daru Island (*7*), illustrating a greater genomic diversity for this lineage. This finding might be an indication of ongoing microevolution, which could further influence transmissibility, acquisition of drug resistance, or severity of disease (*25*).

Our study identified 2 plausible independent episodes of drug-resistant TB transmission to Australia residents in the TSPZ, a finding that would not have been identified by conventional genotyping techniques. Others have shown the superiority of WGS over conventional genotyping tools for resolving transmission (*11*,*26*). Two of the Australia patients with MDR TB were residing in major urban centers in northern Queensland at the time of diagnosis; 1 was living in a residential congregate setting, demonstrating the potential for diffuse community spread. WGS confirmed that the initial isolate noted in the index Australia patient was a lineage 4 isolate with only streptomycin resistance (*gidB*, Leu79Ser) (data not shown). This isolate was unrelated to the other Euro-American isolates identified and different from the subsequent MDR TB Beijing isolate identified, consistent with exogenous reinfection or endogenous reactivation rather than persistent infection with acquired resistance (*27*).

In another study conducted in Europe, WGS was used to investigate MDR TB outbreaks among immigrants and traced transmission routes to strains circulating in northern Somalia or Djibouti (*28*). Although no evidence of transmission to citizens of Europe was noted in that study, our investigation proves the principle of MDR TB cross-border transmission. Our findings highlight the challenge faced by Australia and Papua New Guinea TB control programs to prevent TB transmission through the TSPZ. Over the study period, the number of TB cases among citizens of Australia in the TSPZ was low (18 cases). Most TB notifications on the Australia island communities of the TSPZ were of diagnoses among Papua New Guinea citizens treated in outreach clinics located on the outer Torres Strait Islands close to the Papua New Guinea border. These clinics were closed in 2012, and all Papua New Guinea patients were handed over to the Western Province TB program for treatment, and enhanced TB services were coordinated from Daru Island. This closure and change in services accounts for the sharp fall in notifications in 2012 (Appendix Figure 6).

We observed transmission of a locally evolved Beijing sublineage strain that was associated with resistance to first- and second-line TB drugs. Beijing strains are known to be associated with increased transmissibility (*29*–*31*), drug resistance, treatment failure, and rapid progression to active disease (*32*,*33*). 

Although the World Health Organization estimates a high TB incidence among persons living with HIV in Papua New Guinea (44 cases/100,000 population) (*1*), HIV incidence in our study was low and did not account for the highly successful transmission of Beijing sublineage 2.2.1.1 in this region. In another study in Gulf Province, Papua New Guinea, only 2 HIV-infected persons were identified among 105 TB patients (*34*).

The high proportion (74%) of TB cases among young adults (<35 years of age) in our study suggests ongoing TB transmission and is consistent with the finding of another study that identified 76.6% of pediatric TB notifications in Queensland were from Papua New Guinea residents in the TSPZ (*35*). Further assessment is needed of other risk factors, such as medical conditions (e.g., diabetes mellitus, high blood pressure) and socioeconomic status, that could affect TB transmission.

Excluding ethambutol, we observed a near-perfect correlation between resistance mutations and phenotypic in vitro resistance. One XDR TB isolate had resistance to kanamycin (mycobacterial growth indicator tube system critical concentration 2.5 µg/mL and a microbroth dilution MIC of 10 µg/mL), but no known resistance-conferring mutations were documented. Isolates with low-level kanamycin resistance are generally thought to display resistance to only kanamycin among the aminoglycoside drugs (*36*), but this isolate had a high level of kanamycin resistance. A previous study determined that only 80% of phenotypic kanamycin resistance could be accounted for by known mutations, indicating unexplained mechanisms of kanamycin resistance (*37*). Although the *fabG1*-*inhA* mutation is typically associated with low-level isoniazid resistance, we usually observed high-level isoniazid resistance with this mutation, possibly because of the accompanying *inhA* (p.Ile21Val) mutation (*38*). The *fabG1*-*inhA* (C15T) mutation does not seem to compromise successful spread and has previously been documented in clustered *M. tuberculosis* isolates in South Africa (*39*). One isolate had a *fabG1*-*inhA* (C15T) mutation with a rare *katG* mutation (p.Trp191Arg), which could affect the conformation of the catalase peroxidase protein, considering this mutation is located outside the active binding site of isoniazid (*40*).

Unexpectedly, we observed most rifampin-resistant isolates had mutations in *rpoC*. The fitness cost associated with some drug-resistant mutations can be ameliorated by compensatory mutations (*41*). Most isolates (96.5%) with *rpoC* compensatory mutations demonstrated genomic clustering, signifying their probable role in MDR or XDR TB transmission. In an assessment of MDR TB isolates in Argentina, no evidence was found to support the role of *rpoC* mutations in fitness restoration and increased transmission (*42*). Higher proportions of compensatory mutations in MDR TB outbreaks were reported in Shanghai, China (66%) (*32*), and Samara, Russia (87%) (*11*). More studies are needed to assess the role of compensatory mutations in MDR and XDR strain transmission.

This study had a few limitations. The retrospective study design involved the selection of only 1 isolate per patient, and some of the stored isolates were unable to be studied because of failure to recover from culture. Close social contact between patients with genomically linked isolates could not be confirmed. In other studies, a refined breakdown of transmission patterns was shown with a similar approach that included data on social contacts (*28*,*32*,*43*). Although the current scope of the study comprised mostly Papua New Guinea residents, additional information is needed regarding the evolution and transmission dynamics among all residents in the TSPZ, including drug-susceptible isolates in Australia citizens residing in the TSPZ, to assess the implications of transmission to populations on the Australia mainland.

The combination of genomic and epidemiologic data in this study highlighted the wide distribution of a Beijing sublineage strain associated with MDR and XDR TB in the TSPZ that threatens regional TB control through cross-border transmission. Further investigation of the environmental, sociocultural, and clinical factors that facilitate TB transmission in this region is warranted. Supplementation of regional TB surveillance programs with WGS technology could improve control strategies.

Appendix Additional information on the cross-border movement of highly drug-resistant *Mycobacterium tuberculosis* isolates through the Torres Strait Protected Zone, 2010–2015.
