# Correction: Vol. 25, No. 3

**DOI:** 10.3201/eid2510.C12510

**Published:** 2019-10

**Keywords:** errata, erratum, correction

Figure 2 contained incorrect values in Cross-Border Movement of Highly Drug-Resistant Mycobacterium tuberculosis from Papua New Guinea to Australia through Torres Strait Protected Zone, 2010–2015 (A. Bainomugisa et al.). The corrected figure is provided, and the article has been corrected online (https://wwwnc.cdc.gov/eid/article/25/3/18-1003_article).

