# Supplementary material for: Cross-Border Movement of Highly Drug-Resistant Mycobacterium tuberculosis from Papua New Guinea to Australia through Torres Strait Protected Zone, 2010–2015
Source: Emerg Infect Dis. 2019 Mar;25(3):406–15. doi: 10.3201/eid2503.181003 (PMC6390734; doi:10.3201/eid2503.181003)
Supplement: Appendix — Additional information on the cross-border movement of highly drug-resistant Mycobacterium tuberculosis isolates through the Torres Strait Protected Zone, 2010–2015. [file 18-1003-Techapp-s1.pdf]

Article DOI: https://doi.org/10.3201/eid2503.181003

Cross-Border Movement of Highly Drug-Resistant *Mycobacterium tuberculosis* from Papua New Guinea to Australia through Torres Strait Protected Zone, 2010–2015

Appendix

Appendix Table 1. *Mycobacterium tuberculosis* genes associated with resistance or compensatory mutations (*1*–*13*)

Drug	Genes
Rifampin	*rpoB, rpoC, rpoA, rpoD*
Isoniazid	*fabG1-inhA, inhA, katG, ndh, furA, oxyR, aphC, fadE24, srmR, kasA, mshA*
Ethambutol	*embB, embC, embA, ubiA, embR, iniA, iniC, manB*
Pyrazinamide	*pncA, rpsA, panD*
Streptomycin	*rpsL, gidB, rrs*
Ethionamide	*fabG1-inhA, ethA, ethR*
Ofloxacin	*gyrA, gyrB*
Amikacin	*rrs, whib7, gidB*
Kanamycin	*Rv2417c-eis, whiB7, rrs, gidB*
Capreomycin	*tlyA, whiB7, rrs, gidB*
Paraaminosalicylic acid	*ribD, thyA, dfrA, folC*
Cycloserine	*alr, ddl, cycA*
Bedaquiline	*atpE, Rv0678*
Linezolid	*rrl, rplC*
Delamanid	*fgd1, ddn, fbiA, fbiB, fbiC*

Appendix Table 2. MIRU-24 and MIRU-15 profiles of *Mycobacterium tuberculosis* isolates from Papua New Guinea and Australia citizens residing or previously residing in the Torres Strait Protected Zone, 2010–2015*

MIRU-24 and MIRU-15 profiles	No. patients
244252352644425163353824†	12
2_42423_2_4__25163_6___4	6
2_42423_2_4__25163_5___4	57
2_42423_2_4__25163_4___4	2
2_42431_2_3__25153_3___2	5
2_42132_2_3__26153_3___2	7
2_43122_1_4__22123_5___3	1
2_42543_2_2__26153_2___2	2
2_42232_2_3__26211_5___3	1
3_43233_2_4__25153_2___2	2
214213222433226153334502	3
Missing	6
Total	104

*Underscore indicates loci not assessed. Loci were analyzed in the following order: 154, 424, 577, 580, 802, 960, 1644, 1955, 2059, 2163b, 2165, 2347, 2401, 2461, 2531, 2687, 2996, 3007, 3171, 3192, 3690, 4052, 4156, and 4348. MIRU, mycobacterial interspersed repetitive unit.
†All the isolates from the Australia residents had profile 244252352644425163353824.

Appendix Table 3. *Mycobacterium tuberculosis* lineages present in Papua New Guinea and Australia citizens residing or previously residing in the Torres Strait Protected Zone, 2010–2015*

Lineage	Position, bp	Allele change	Codon change	Gene name	Coding	Spoligotype	No. strains
2.2.1.1	4248115	C→T	GAC→GAT	*embB*	Synonymous	Beijing	83
4.1.2.1	107794	C→T	GCC→GCT	*fcoT*	Synonymous	X-type	1
4.2.1	783601	A→C	AGG→CGG	*fusA1*	Synonymous	TUR	1
4.3.1	615614	C→A	GCC→GCA	*hemL*	Synonymous	LAM	2
4.4.1.1	355181	G→A	AAG→AAA	*mycP3*	Synonymous	S-type	2
4.8	3836739	G→A	GAC→GAT	*groEL1*	Synonymous	mainly T	7
4.9	1759252	G→T	TCG→TCT	*frdA*	Synonymous	H37Rv-like	8

*Classification according to Coll et al (*14*).

Appendix Table 4. In vitro drug susceptibility of *Mycobacterium tuberculosis* isolates of Papua New Guinea and Australia citizens residing or previously residing in the Torres Strait Protected Zone, 2010–2015

Drug	Susceptible	Resistant
Rifampin	69	35
Isoniazid*	53	20
Isoniazid†		31
Ethambutol	94	10
Pyrazinamide	85	19
Streptomycin	53	51
Ethionamide‡	1	34
Ofloxacin‡	32	3
Amikacin‡	34	1
Kanamycin‡	33	2
Capreomycin‡	34	1
Paraaminosalicyclic acid‡	35	0
Cycloserine‡	35	0

*Critical concentration of 0.1 µg/mL in mycobacterial growth indicator tube system.
†Critical concentration of 0.4 µg/mL in mycobacterial growth indicator tube system.
‡Drugs tested if isolate was rifampin resistant or on clinical request.

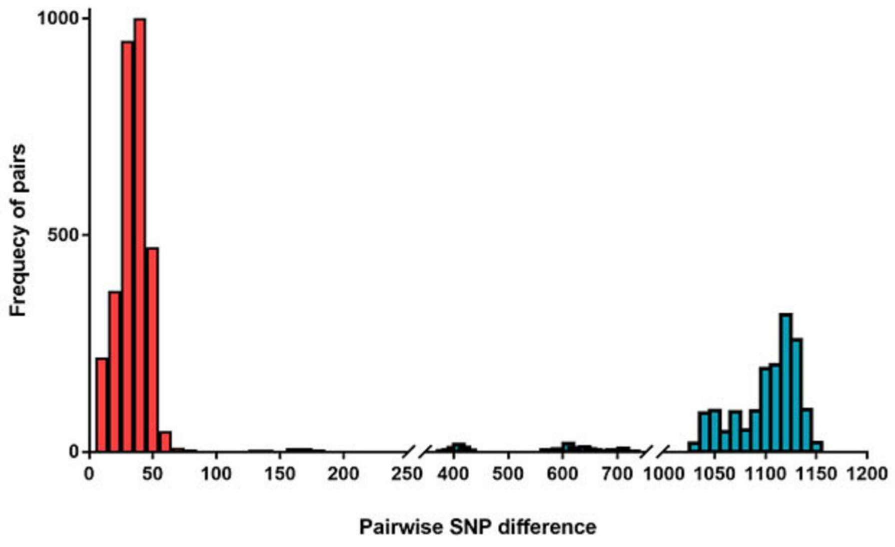

Appendix Figure 1. Pairwise SNP difference among all cross-border isolates in the Torres Strait Protected Zone, 2010–2015. Red bars represent same mycobacterial interspersed repetitive unit (MIRU) profile, blue different MIRU profile, and purple no MIRU profile. SNP differences between all possible pairs within the data set was bimodal, with 2 large peaks representative of the 2 lineages. SNP, single-nucleotide polymorphism.

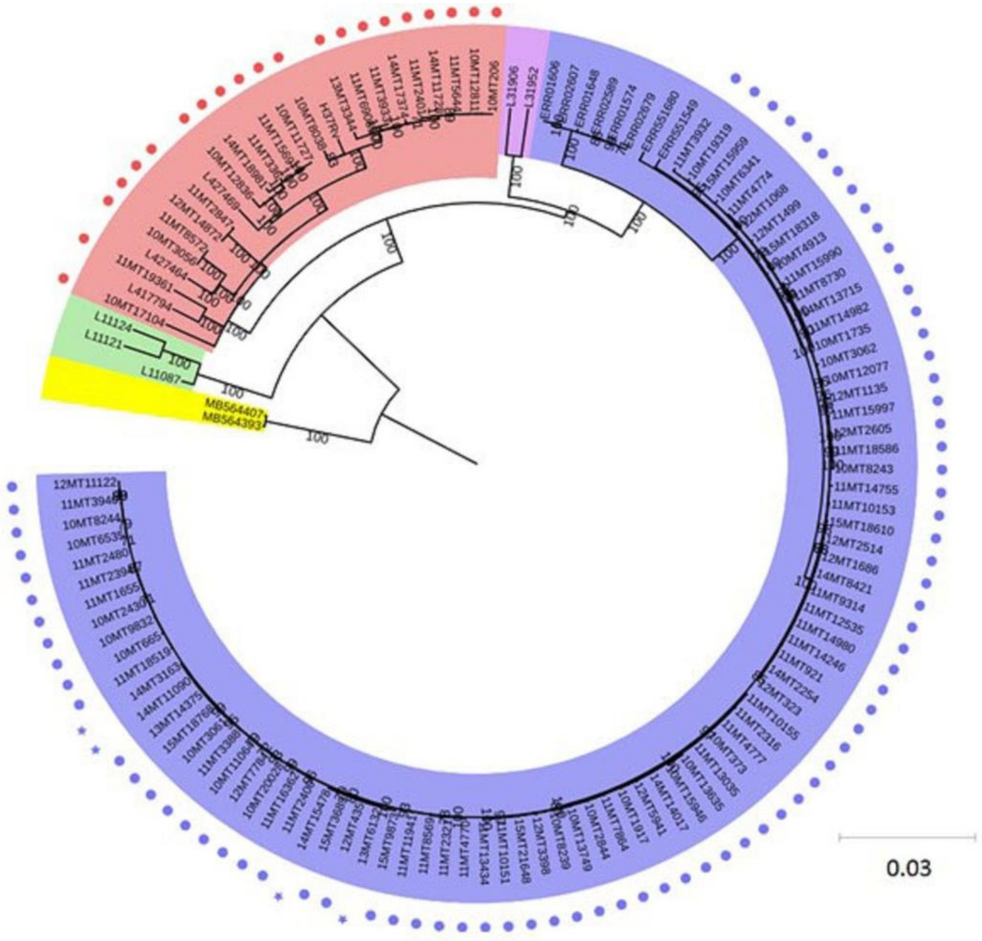

Appendix Figure 2. Maximum-likelihood tree of *Mycobacteriun tuberculosis* isolates from Papua New Guinea (circles) and Australia (stars) citizens residing or previously residing in the Torres Strait Protected Zone, 2010–2015, including representative genomes and rooted with *M. bovis.* Lineages are indicated by color: *M. bovis* (yellow), *M. tuberculosis* Indo-Oceanic (light green), *M. tuberculosis* Euro-American (red), *M. tuberculosis* East African-Indian (magenta), and *M. tuberculosis* East Asian (lavender). Numbers on branches indicate bootstrap values (70%–100%). Scale bar indicates nucleotide substitutions per base pair.

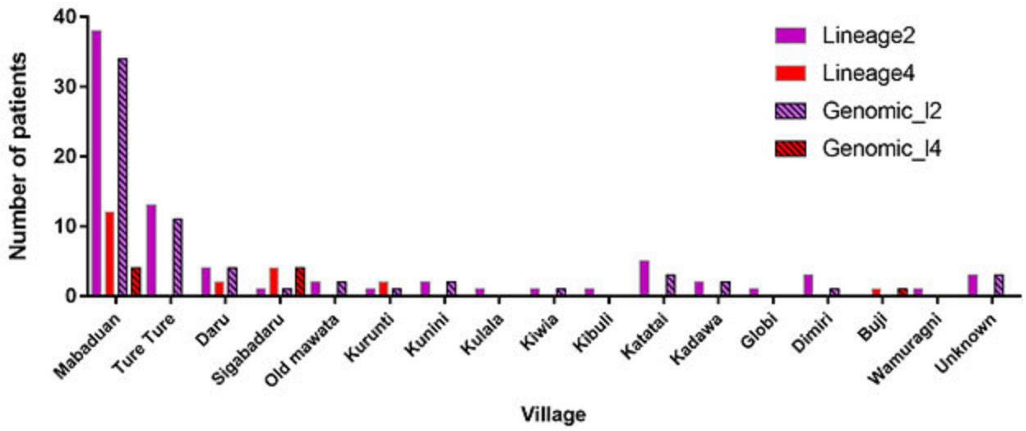

Appendix Figure 3. Number of patients infected with specific *Mycobacterium tuberculosis* lineage strains in each village or town of the Torres Strait Protected Zone, 2010–2015. Lineage 2 (Beijing sublineage, magenta), genomic clusters of lineage 2 (hatched lines and magenta), lineage 4 (red), and genomic clusters of lineage 4 (hatched lines and red) are indicated.

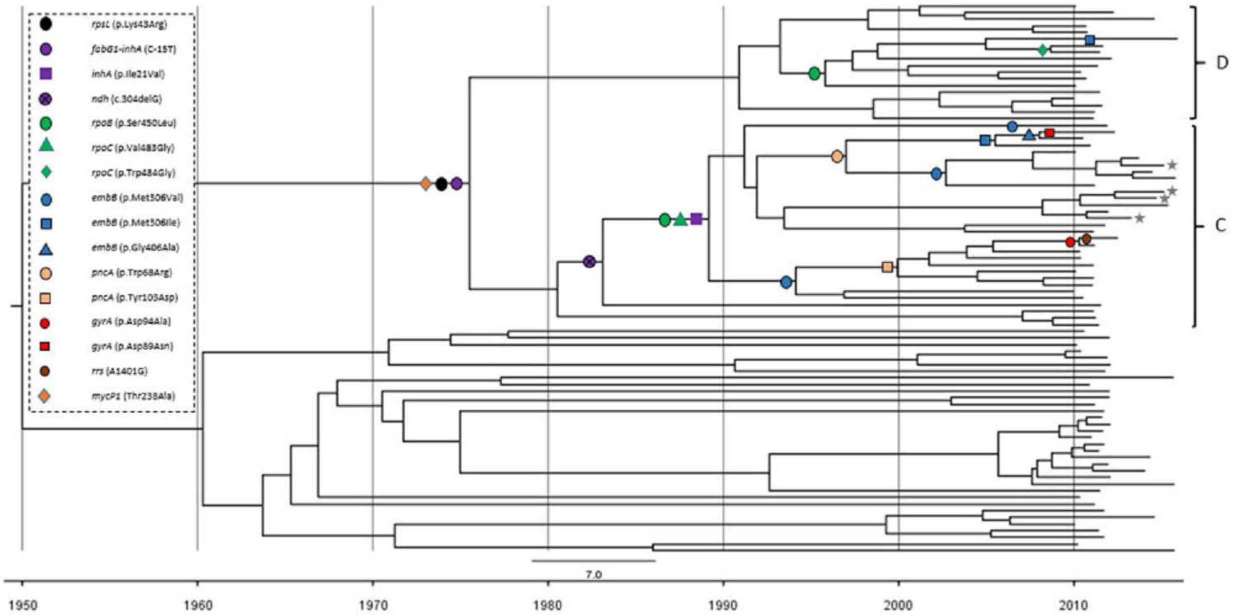

Appendix Figure 4. Molecular clock tree of 83 Beijing sublineage isolates with parsimonious mapping of known resistance conferring mutations, Torres Strait Protected Zone, 2010–2015. Stars indicate isolates from Australia residents in the Torres Strait. Clade C and D are previously described (*15*).

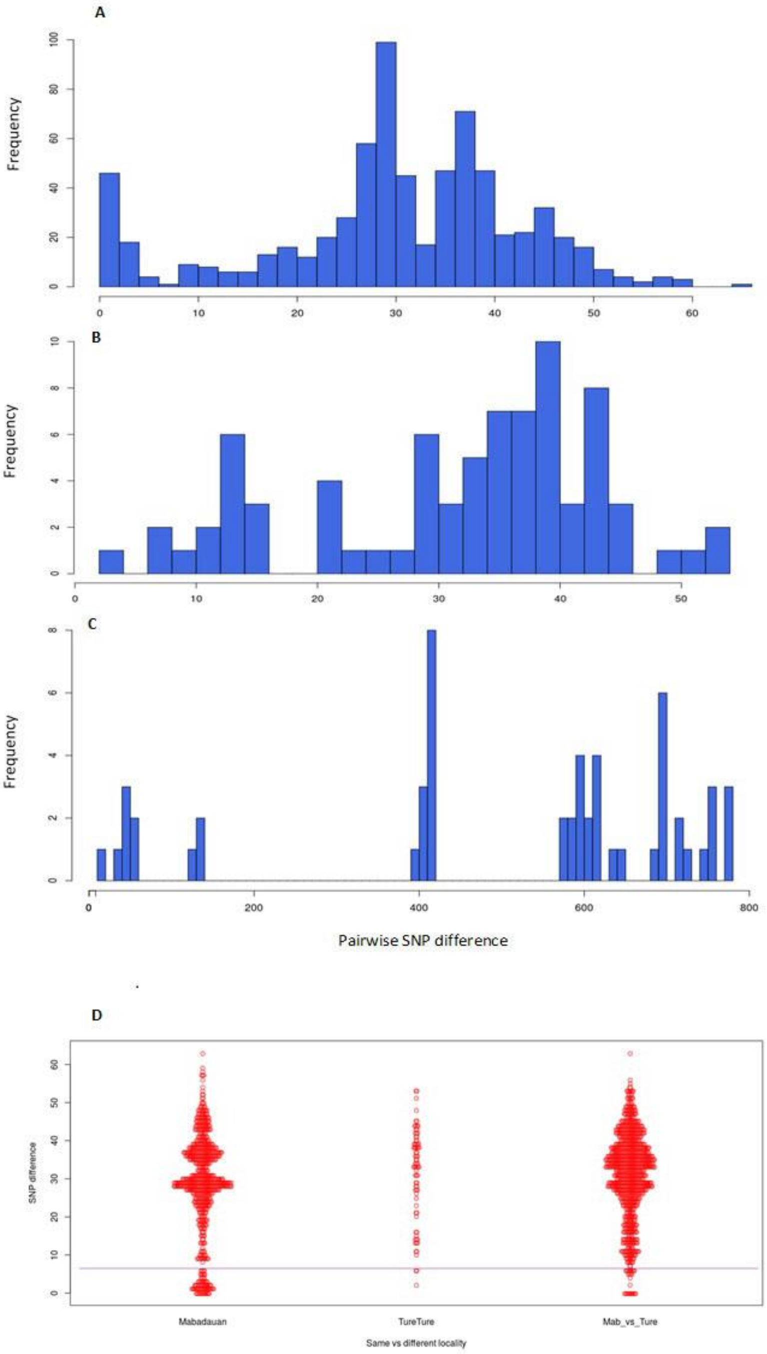

Appendix Figure 5. Pairwise SNP differences among different lineages identified in same locality, Torres Strait Protected Zone, 2010–2015. A) Beijing sublineage (Mabadauan); B) Beijing sublineage (Ture Ture); and C) Euro-American (Mabadauan). D) Comparison of Beijing strains from the same and different localities. Line indicates the threshold to distinguish plausible transmission links (8 SNP differences). SNP, single-nucleotide polymorphism.

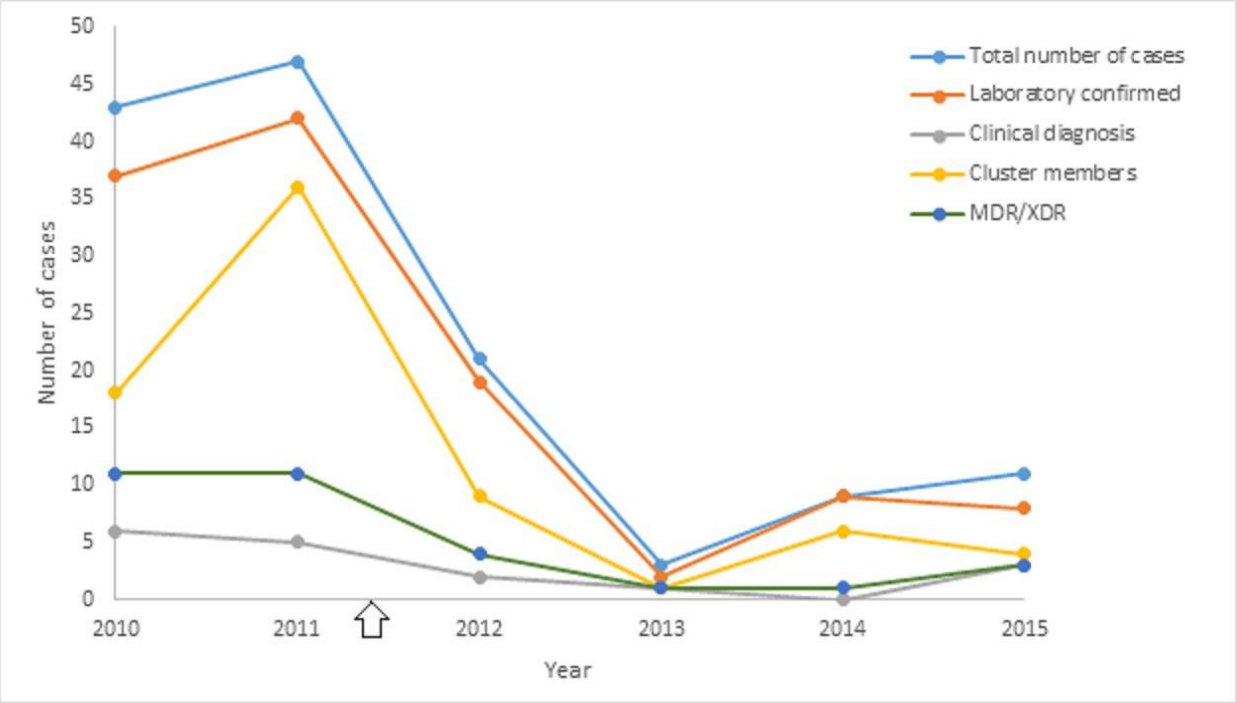

Appendix Figure 6. Trend of notified tuberculosis cases among patients in the Torres Strait Protected Zone, 2010–2015. Data provided by the Epidemiology and Research Unit, Queensland Health (Brisbane, Queensland, Australia). Arrow indicates the commencing of the closure of tuberculosis clinics on the outer islands; patient handover to Papua New Guinea health authorities was completed by mid-2012 through a series of bipartisan clinics.

References

1. Ramaswamy SV, Dou SJ, Rendon A, Yang Z, Cave MD, Graviss EA. Genotypic analysis of multidrug-resistant *Mycobacterium tuberculosis* isolates from Monterrey, Mexico. J Med Microbiol. 2004;53:107–13. PubMed http://dx.doi.org/10.1099/jmm.0.05343-0

2. Bloemberg GV, Keller PM, Stucki D, Trauner A, Borrell S, Latshang T, et al. Acquired resistance to bedaquiline and delamanid in therapy for tuberculosis. N Engl J Med. 2015;373:1986–8. PubMed http://dx.doi.org/10.1056/NEJMc1505196

3. Comas I, Borrell S, Roetzer A, Rose G, Malla B, Kato-Maeda M, et al. Whole-genome sequencing of rifampicin-resistant *Mycobacterium tuberculosis* strains identifies compensatory mutations in RNA polymerase genes. Nat Genet. 2012;44:106–10. PubMed http://dx.doi.org/10.1038/ng.1038

4. Jnawali HN, Yoo H, Ryoo S, Lee KJ, Kim BJ, Koh WJ, et al. Molecular genetics of *Mycobacterium tuberculosis* resistant to aminoglycosides and cyclic peptide capreomycin antibiotics in Korea. World J Microbiol Biotechnol. 2013;29:975–82. PubMed http://dx.doi.org/10.1007/s11274-013-1256-x

5. Mathys V, Wintjens R, Lefevre P, Bertout J, Singhal A, Kiass M, et al. Molecular genetics of para-aminosalicylic acid resistance in clinical isolates and spontaneous mutants of *Mycobacterium tuberculosis*. Antimicrob Agents Chemother. 2009;53:2100–9. PubMed http://dx.doi.org/10.1128/AAC.01197-08

6. Köser CU, Bryant JM, Becq J, Török ME, Ellington MJ, Marti-Renom MA, et al. Whole-genome sequencing for rapid susceptibility testing of *M. tuberculosis*. N Engl J Med. 2013;369:290–2. PubMed http://dx.doi.org/10.1056/NEJMc1215305

7. Manson AL, Cohen KA, Abeel T, Desjardins CA, Armstrong DT, Barry CE III, et al.; TBResist Global Genome Consortium. Genomic analysis of globally diverse *Mycobacterium tuberculosis* strains provides insights into the emergence and spread of multidrug resistance. Nat Genet. 2017;49:395–402. PubMed http://dx.doi.org/10.1038/ng.3767

8. Sandgren A, Strong M, Muthukrishnan P, Weiner BK, Church GM, Murray MB. Tuberculosis drug resistance mutation database. PLoS Med. 2009;6:e1000002. PubMed http://dx.doi.org/10.1371/journal.pmed.1000002

9. Zheng J, Rubin EJ, Bifani P, Mathys V, Lim V, Au M, et al. Para-aminosalicylic acid is a prodrug targeting dihydrofolate reductase in *Mycobacterium tuberculosis*. J Biol Chem. 2013;288:23447–56. PubMed http://dx.doi.org/10.1074/jbc.M113.475798

10. Zaunbrecher MA, Sikes RD Jr, Metchock B, Shinnick TM, Posey JE. Overexpression of the chromosomally encoded aminoglycoside acetyltransferase *eis* confers kanamycin resistance in *Mycobacterium tuberculosis*. Proc Natl Acad Sci U S A. 2009;106:20004–9. PubMed http://dx.doi.org/10.1073/pnas.0907925106

11. Dheda K, Gumbo T, Maartens G, Dooley KE, McNerney R, Murray M, et al. The epidemiology, pathogenesis, transmission, diagnosis, and management of multidrug-resistant, extensively drug-resistant, and incurable tuberculosis. Lancet Respir Med. 2017;S2213-2600(17)30079-6. PubMed

12. Bloemberg GV, Keller PM, Stucki D, Trauner A, Borrell S, Latshang T, et al. Acquired resistance to bedaquiline and delamanid in therapy for tuberculosis. N Engl J Med. 2015;373:1986–8. PubMed http://dx.doi.org/10.1056/NEJMc1505196

13. Zhang S, Chen J, Cui P, Shi W, Zhang W, Zhang Y. Identification of novel mutations associated with clofazimine resistance in *Mycobacterium tuberculosis*. J Antimicrob Chemother. 2015;70:2507–10. PubMed http://dx.doi.org/10.1093/jac/dkv150

14. Coll F, McNerney R, Guerra-Assunção JA, Glynn JR, Perdigão J, Viveiros M, et al. A robust SNP barcode for typing *Mycobacterium tuberculosis* complex strains. Nat Commun. 2014;5:4812. PubMed http://dx.doi.org/10.1038/ncomms5812

15. Bainomugisa A, Lavu E, Hiashiri S, Majumdar S, Honjepari A, Moke R, et al. Multi-clonal evolution of multi-drug-resistant/extensively drug-resistant *Mycobacterium tuberculosis* in a high-prevalence setting of Papua New Guinea for over three decades. Microb Genom. 2018;4. PubMed http://dx.doi.org/10.1099/mgen.0.000147
